# Wafer Scale III‐Nitride Deep‐Ultraviolet Vertical‐Cavity Surface‐Emitting Lasers Featuring Nanometer‐Class Control of Cavity Length

**DOI:** 10.1002/advs.202520405

**Published:** 2025-12-21

**Authors:** Chen Ji, Jiaming Wang, Fujun Xu, Lisheng Zhang, Jing Lang, Ziyao Zhang, Fuyun Tan, Chengzhi Ji, Junchuan Zhang, Erfei Zhang, Xiangning Kang, Zhixin Qin, Guangxu Ju, Jiejun Wu, Xuelin Yang, Ning Tang, Xinqiang Wang, Weikun Ge, Bo Shen

**Affiliations:** ^1^ State Key Laboratory of Artificial Microstructure and Mesoscopic Physics School of Physics Peking University Beijing China; ^2^ School of Integrated Circuits Peking University Beijing China; ^3^ Beijing SinoGaN Semiconductor Technology Co., Ltd. Beijing China; ^4^ Nano‐optoelectronics Frontier Center of Ministry of Education Peking University Beijing China; ^5^ Collaborative Innovation Center of Quantum Matter Beijing China

**Keywords:** III‐nitrides, cavity length, DUV VCSELs, self‐terminated etching

## Abstract

AlGaN‐based deep‐ultraviolet vertical‐cavity surface‐emitting lasers (DUV VCSELs) have shown a great application potential in optical atomic clocks, maskless photolithography, etc. Nevertheless, the uncontrolled cavity length‐induced detuning issue, i.e., the difference between the resonance wavelength and gain peak, severely impairs the device performance. Herein, a DUV‐VCSEL strategy featuring the uniform nanometer‐class control of the cavity length in a 4‐in wafer is proposed in the DUV framework based on GaN templates, which ensures the wafer‐scale removal of sapphire substrates by laser lift‐off, and then provides space for the subsequent deposition of dielectric distributed Bragg reflector (DBR). It is more significant that the strategy brings about a GaN/AlGaN sharp interface with an Al composition difference up to 80%, whereby self‐terminated etching with an ultrahigh selectivity of 100:1 is achieved. The cavity length is hence accurately determined by epitaxy itself instead of the fabrication process, so as to minimize the detuning. As such, 285.6‐nm optically pumped DUV VCSELs with double dielectric DBRs are fabricated, exhibiting a record low threshold of 0.38 MW cm^−2^ and a narrow linewidth of 0.11 nm. What's more, the lasing wavelength varies within 1.9 nm across the 4‐in wafer, indicating a cavity length variation of only 0.81%.

## Graphical abstract

A DUV‐VCSEL strategy featuring the nanometer‐class control of the cavity length is proposed in the DUV optoelectronic framework based on GaN templates. After the sapphire removal, a self‐terminated etching technology is developed, whereby the cavity length can be accurately determined by epitaxy instead of the fabrication process. As such, a record low threshold is realized in 285.6‐nm optically pumped DUV VCSELs.

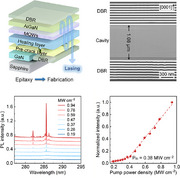

## 1 Introduction

Vertical‐cavity surface‐emitting lasers (VCSELs) have attracted much attention owing to their advantages of circular far field distribution, single longitudinal mode emission, and 2D integration capability, leading to a rapidly growing billion‐dollar market in data communication, sensing, and display [1, 2, 3]. From III‐arsenides, III‐phosphides to III‐nitrides, the lasing wavelength blue‐shifts from infrared/red [4, 5, 6] to blue/ultraviolet (UV) [7, 8, 9], which further expands the application scenarios to high‐resolution 3D nanoprinting, maskless photolithography, and miniature atomic clocks [10, 11, 12]. Nevertheless, a serious challenge for III‐nitride VCSELs is the realization of distributed Bragg reflectors (DBRs) with a high reflectivity. Unlike the mature AlGaAs/GaAs DBRs, the lower refractive index difference and larger lattice mismatch between III‐nitrides make it quite hard to achieve a crack‐free epitaxial DBR (e.g., AlN/GaN DBR) with a high reflectivity as well as a wide stopband [13]. The situation is even worse when it enters the UV band, since AlGaN with high Al composition is essential in AlGaN/Al(Ga)N DBRs to avoid the light absorption, which further reduces the refractive index difference. An alternative and effective solution is the adoption of dielectric DBRs, e.g., TiO_2_/SiO_2_ [14], ZrO_2_/SiO_2_ [15], Ta_2_O_5_/SiO_2_ [16], and HfO_2_/SiO_2_ [17]. In order to furthest minimize the mirror losses and thus decrease the threshold, VCSELs with double dielectric DBRs is proposed, which, however, brings about new challenges: (i) how to remove the substrate without cracks and subsequently obtain a smooth exposed surface for the deposition of the second DBR [18, 19, 20]; (ii) how to precisely control the cavity length after the substrate removal, so as to reduce the detuning between the resonance wavelength and gain peak [21].

In terms of the substrate removal, a series of approaches have been proposed, including laser lift‐off (LLO) [9, 18, 20], chemical/electrochemical lift‐off [8, 22, 23], and etch thinning [19]. Wherein, LLO is the most promising candidate by comprehensively considering the productivity, yield, and cost, hence widely adopted in GaN‐based visible optoelectronics [24, 25, 26]. It is, however, fairly hard to apply LLO in AlGaN‐based DUV devices grown on AlN templates. The key issue is that the precipitated Al from AlN decomposition is rigid, resulting in serious cracking of the lifted‐off epilayers [27]. A ground‐breaking DUV optoelectronic framework featuring structural stacking on GaN templates has been demonstrated in our previous work, where the 4‐inch sapphire substrate can be entirely removed by LLO without cracks, and then the wafer‐scale fabrication of 280‐nm vertical injection DUV light‐emitting diodes is realized [28]. Definitely, the framework should also be applicable to the fabrication of DUV VCSELs.

It is worth noting that the substrate removal by LLO brings about a rough exposed surface, which will cause severe optical scattering losses in the cavity and then make lasing difficult [29]. Chemical mechanical polishing (CMP) is hence widely employed to smooth the surface as well as control the cavity length [9, 30, 31], although it is not really satisfactory. On the one hand, the lifted‐off epilayer generally curls owing to the residual stress; consequently, the uniformity of the cavity length after CMP is quite poor with a variation of hundreds of nanometers in a large‐size wafer [9], much worse than that by electrochemical lift‐off [23]. On the other hand, the thickness control of CMP cannot meet the precision requirement of the cavity, wherein the uncontrolled cavity length will result in the detuning between the resonance wavelength and gain peak, and then lead to an evident increase in the threshold [21]. This issue has long plagued III‐nitride VCSELs, from InGaN‐based visible to AlGaN‐based UV ones. Besides CMP, an approach of photo‐assisted chemical etching is proposed to smooth the exposed surface, by which a measure of reduction in the roughness is achieved [32]. The high chemical reactivity of the N‐polar surface in aqueous acid/alkali, however, plays a negative role and restricts the effect of this approach.

In this work, a DUV‐VCSEL strategy featuring the nanometer‐class control of the cavity length is proposed in the DUV optoelectronic framework based on GaN templates, which ensures the LLO removal of the sapphire substrates for the deposition of dielectric DBR, meanwhile maintaining a high radiative recombination efficiency for lasing. After the sapphire removal, a self‐terminated dry etching technology is developed, whereby the cavity length can be accurately determined by epitaxy instead of the fabrication process, so as to minimize the detuning between the resonance wavelength and gain peak. Moreover, the etching brings about a smooth surface to reduce the optical scattering losses at the cavity/DBR interface. As such, a record low threshold 0.38 MW cm^−2^ is realized in 285.6‐nm optically pumped DUV VCSELs with double dielectric DBRs, and a cavity length variation of only 0.81% within a total length of 1.08 µm is demonstrated across the 4‐in wafer.

## 2 Results and Discussion

### 2.1 Epitaxy and Optical Properties of the Cavity

**FIGURE 1:**
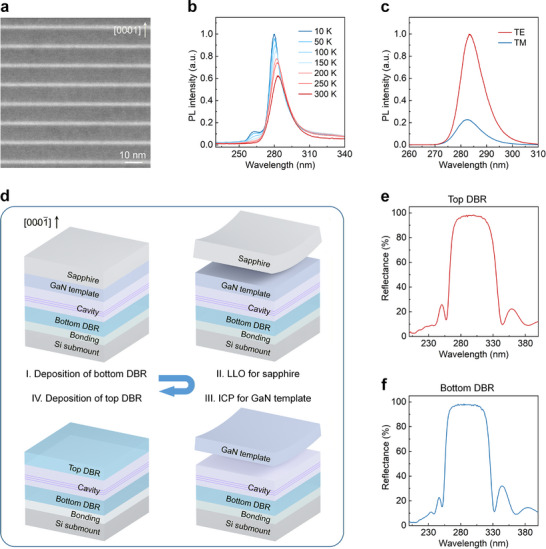
Epitaxy and fabrication of DUV VCSELs. (a) Cross‐sectional HAADF‐STEM image of MQWs in the cavity. (b) Temperature‐dependent PL spectra of MQWs in Panel a. (c) Polarization‐dependent PL spectra of MQWs in Panel a. (d) Schematic illustration of the fabrication of DUV VCSELs with double dielectric DBRs. (e,f) Reflection spectra of the top and bottom DBRs, respectively.

The epitaxy and fabrication of DUV VCSELs are outlined in Figure 1. Our proposed DUV framework is adopted for the sake of removing the sapphire substrate, and a 9λ cavity for DUV lasing (∼280 nm) is grown on GaN templates. The cavity consists of the decoupling structure (a 90‐nm‐thick Al_0.8_Ga_0.2_N pre‐crack layer and an 870‐nm‐thick Al_0.65_Ga_0.35_N healing layer) [28], 8‐pair Al_0.5_Ga_0.5_N/Al_0.37_Ga_0.63_N multiple quantum wells (MQWs), as well as a 45‐nm‐thick Al_0.5_Ga_0.5_N capping layer in sequence. Wherein the position of MQWs is thoroughly designed. On the one hand, both the pre‐crack and healing layers in the decoupling structure are thick enough to fully release the lattice‐mismatch‐induced strain between the GaN template and MQWs, and subsequently rebuild a flat surface for MQWs; meanwhile on the other hand, the MQWs are placed on an optical antinode of the standing wave according to the theoretical calculations as shown below, which can help to reduce the threshold [1].

Figure 1a presents the high‐angle annular dark‐field scanning transmission electron microscopy (HAADF‐STEM) image of MQWs, where the thicknesses of the barriers and wells are determined to be 9 nm and 1.9 nm, respectively. The radiative recombination efficiency (RRE), as one of the decisive factors for lasing, is further evaluated via the temperature‐dependent photoluminescence (PL) measurements from 10 to 300 K (Figure 1b). Assuming the non‐radiative recombination centers are frozen at cryogenic temperature (10 K), the MQWs exhibit a room‐temperature RRE of 71.5%, almost the same as our previous report [28]. Moreover, the optical polarization of MQWs is characterized by polarization‐dependent PL, where the transverse‐electric (TE) and transverse‐magnetic (TM) polarized light is separately collected as shown in Figure 1c. It is found that the TE light hugely dominates, and the degree of polarization (DOP) is estimated as 60.6%, assuring the feasibility of the surface‐emitting lasing.

The fabrication of DUV VCSELs starts with the bottom HfO_2_/SiO_2_ DBR deposited on the as‐grown surface, followed by the wafer bonding and LLO of the sapphire substrate. The subsequent removal of the exposed GaN template is the key to precisely controlling the cavity length. An inductively coupled plasma (ICP) etching process with ultrahigh selectivity is then developed, by which the etching front self‐terminates at the interface between GaN and Al_0.8_Ga_0.2_N (the pre‐crack layer). In other words, the cavity length can be determined by epitaxy instead of etching, hence significantly enhancing the precision and uniformity of the cavity length. Meanwhile, the self‐terminated etching brings about a smooth surface as shown below, which lays a good foundation for the deposition of the top HfO_2_/SiO_2_ DBR. The reflection spectra of the top and bottom DBRs are recorded in Figure 1e,f, respectively, and the corresponding transmission spectra can be found in Figure S1. Both DBRs show a high reflectivity exceeding 96% within the wavelength range of 280–300 nm, consistent with the theoretical expectations.

### 2.2 Self‐Terminated Etching of N‐polar GaN Over Al_0.8_Ga_0.2_N

It should be noted that the exposed GaN surface after the LLO removal of sapphire is N‐polar, exhibiting greater chemical reactivity in comparison with the metal‐polar one [33]. As a consequence, it is more difficult to precisely control the cavity length by etching; worse still, the realization of a smooth surface for the top DBR seems to be an illusion. Fortunately, an AlGaN layer with an extremely high Al composition of 80% (the pre‐crack layer) is adjacent to the GaN template (Figure S2), making it possible to realize a highly selective etching to address these issues.

**FIGURE 2:**
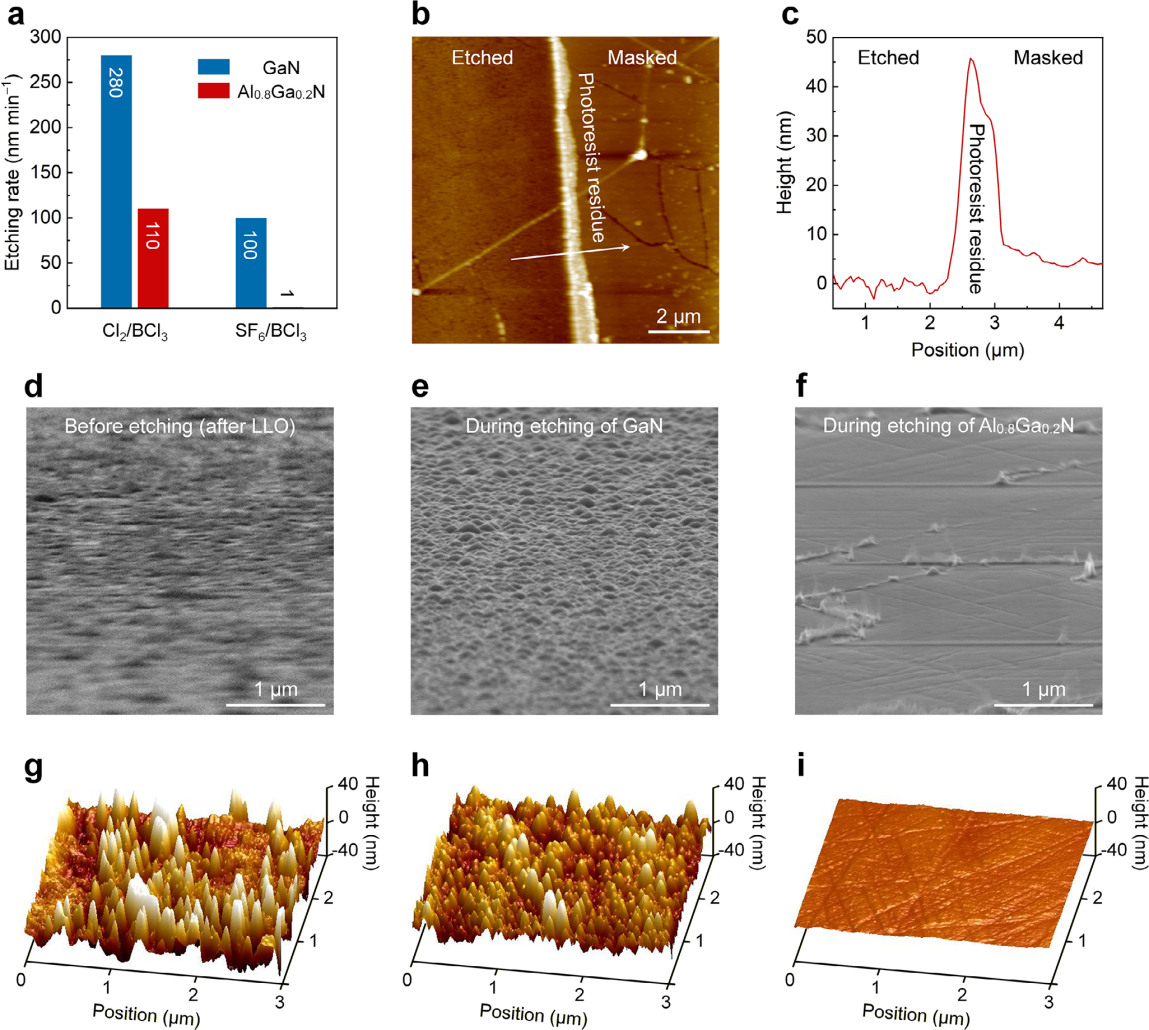
Self‐terminated etching of N‐polar GaN over Al_0.8_Ga_0.2_N. (a) Etching rates of N‐polar GaN and Al_0.8_Ga_0.2_N in the Cl_2_/BCl_3_ and SF_6_/BCl_3_ mixtures. (b) The AFM image of the etched Al_0.8_Ga_0.2_N for 4 min in SF_6_/BCl_3_ mixtures, where the right half is masked by photoresist during etching. (c) The line scan of relative height along the white arrow in Panel b. (d,e,f) Surface morphology of the exposed GaN surface (after LLO), etched GaN, and etched Al_0.8_Ga_0.2_N characterized by SEM, respectively. (g,h,i) Surface morphology by AFM corresponding to Panels d, e, and f, respectively.

Herein, ICP etching is performed in both chlorine‐ and fluorine‐based chemistry, and the etching rates of GaN and Al_0.8_Ga_0.2_N (Figure 2a) are evaluated by atomic force microscopy (AFM), where photoresist is employed as the mask of the unetched region. It is demonstrated that in comparison with Cl_2_/BCl_3_ mixtures, the etching remarkably decelerates in SF_6_/BCl_3_ ones, since the fluorides of Ga and Al have much higher boiling/sublimation points than the chlorides [34]. More importantly, the formation of nonvolatile AlF_x_ almost terminates the Al_0.8_Ga_0.2_N etching with a quite slow rate of only 1 nm min^−1^, leading to an ultrahigh selectivity of 100:1 for N‐polar GaN relative to Al_0.8_Ga_0.2_N. Figure 2b presents the AFM image of the etched Al_0.8_Ga_0.2_N for 4 min in SF_6_/BCl_3_ mixtures, where the etched and masked regions can be easily distinguished by the boundary of the photoresist residue. To determine the etching rate, a line scan of the relative height is performed along the white arrow across the boundary. The etched depth is then determined to be ∼4 nm in Figure 2c; as such, the Al_0.8_Ga_0.2_N etching rate in SF_6_/BCl_3_ mixtures is 1 nm min^−1^.

Such high etching selectivity greatly benefits the precise control and repeatability of the cavity length during the fabrication of DUV VCSELs. Moreover, it can bring about a smooth surface for the subsequent DBR. The surface morphology before/during etching is characterized by scanning electron microscopy (SEM) and AFM, as shown in Figures 2d–i. A typical rough surface is present in the lifted‐off epilayer in Figure 2d, attributed to the spot‐to‐spot irradiation in the LLO process; meanwhile, in a scan area of 3 × 3 µm^2^ by AFM (Figure 2g), dense and whisker‐like microcolumns are observed as reported [35], leading to a root‐mean‐square (RMS) roughness of 12.8 nm. This morphology is almost inherited during GaN etching (Figure 2e,h), since there is no etching selectivity inside the GaN template. Until the etching front reaches the GaN/Al_0.8_Ga_0.2_N interface, exposed Al_0.8_Ga_0.2_N in the valley self‐terminates the process, while residual GaN is still rapidly etched. As a consequence, the surface gradually flattens out, and a roughness of 0.7 nm is eventually achieved (Figure 2i). It is worth noting that small protrusions can be observed in Figure 2f, which are regularly distributed along some straight lines. These lines actually correspond to the pre‐cracks, and the protrusions are AlGaN grown in the pre‐cracks during healing (Figure S3). In consideration of the quite small surface coverage of pre‐cracks, it is believed that these protrusions have less effect on the subsequent deposition of the top DBR.

**FIGURE 3:**
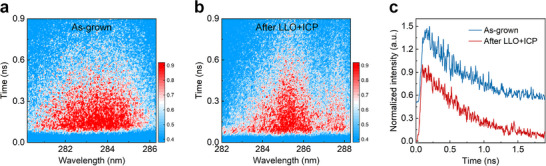
Impact of LLO and etching on the optical properties of MQWs. (a,b) Time‐resolved PL at room temperature for MQWs before and after LLO/ICP, respectively. (c) PL decay profiles at the peak wavelengths in Panels a and b.

Prior to the deposition of the top DBR, the impacts of LLO on the optical properties of MQWs should be revealed, since the laser reaching MQWs during LLO may result in the formation of additional defects for non‐radiative recombination [36, 37]. Herein, the fluence of the KrF excimer laser employed in the LLO process is decreased as much as possible, so as to reduce the potential damage. Time‐resolved PL measurements at room temperature are further carried out to investigate the lifetime of carriers in MQWs [38], which can quantify the variation of the non‐radiative recombination lifetime (i.e., defects) between as‐grown and processed MQWs. According to the images recorded by a streak camera (Figure 3a,b), a redshift of the peak wavelength is observed from 283 nm (as‐grown) to over 285 nm (processed), attributed to the variation of residual strain in the epilayer owing to the processes of bonding and LLO (details in Figures S4 and S5) [39]. Notably, the PL decay profiles at the peak wavelengths are shown in Figure 3c, where the carrier lifetime is extracted to be ∼660 ps for both samples. This indicates that the LLO and ICP processes in this study do not introduce additional point defects into MQWs, assuring the cavity quality in the DUV VCSELs.

### 2.3 Performance of the DUV VCSELs

**FIGURE 4:**
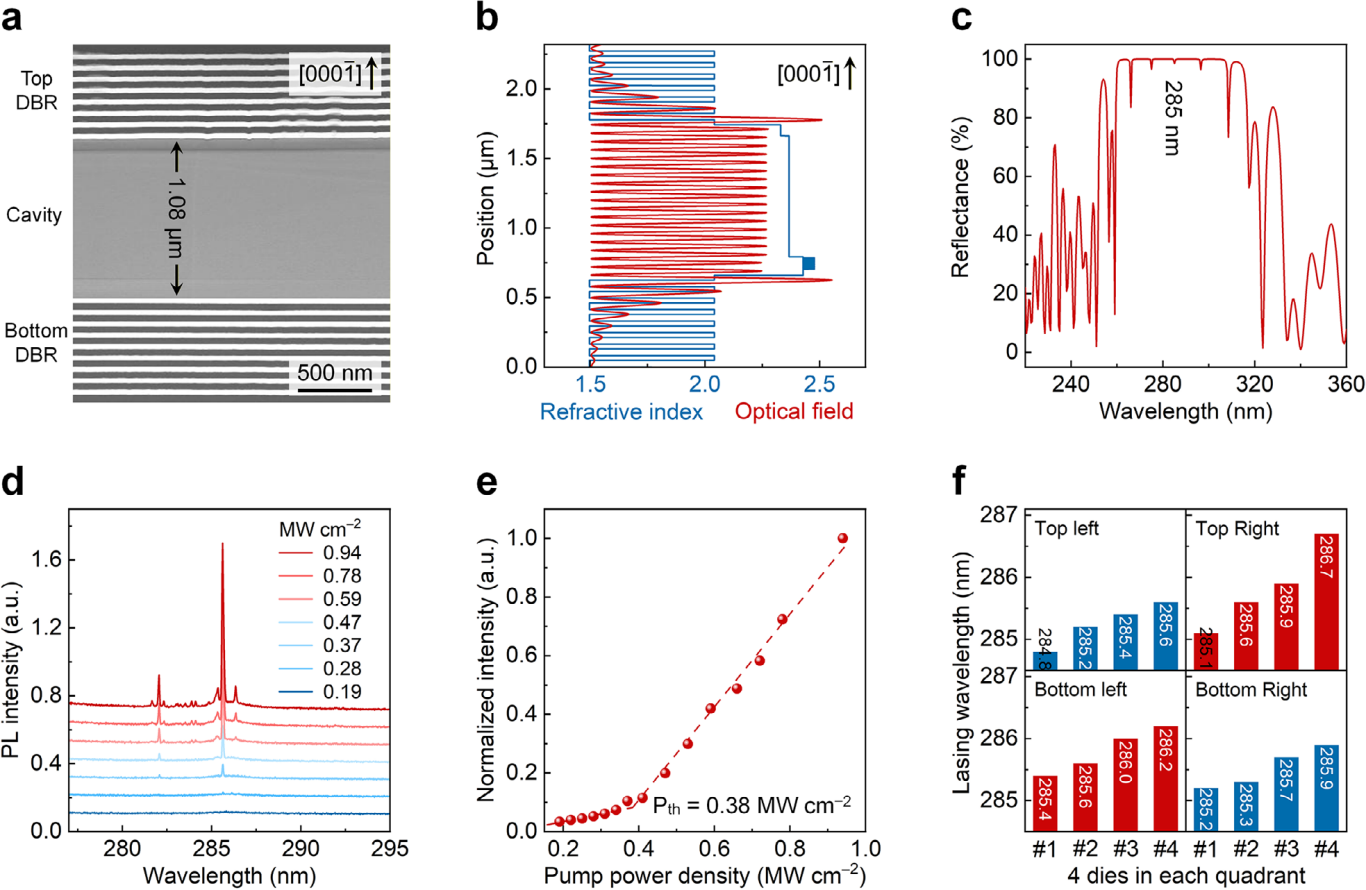
Performance of DUV VCSELs. (a) Cross‐sectional HAADF‐STEM image of the device. (b) Calculation result of the optical field inside the cavity. (c) Calculated cavity reflection spectrum. (d) PL emission spectra at room temperature under different optical pump power densities. (e) Dependence of the integrated intensity of the 285.6‐nm stimulated peak on the pump power density. (f) Lasing wavelengths of 4 random dies in each quadrant of the 4‐in wafer.

The 4‐in wafer‐scale fabrication of DUV VCSELs is further accomplished along with the deposition of the top DBR. According to the cross‐sectional HAADF‐STEM measurement shown in Figure 4a, the cavity length is determined to be 1.08 µm, almost equivalent to the sum of the epitaxial thickness from the Al_0.8_Ga_0.2_N pre‐crack layer to the Al_0.5_Ga_0.5_N capping one. That demonstrates the nanometer‐class control of the cavity length through the combination of LLO and self‐terminated ICP etching. Also, it is found that both the top and bottom DBRs present flat interfaces, attributed to the aforementioned smooth etched and as‐grown surfaces, respectively. The longitudinal optical field inside the DUV‐VCSEL structure is then calculated by the transfer matrix method, as shown in Figure 4b. On the one hand, most of the QWs align with the optical antinode as designed to obtain a great gain; meanwhile, both interfaces between cavity and top/bottom DBR are placed at the optical node, beneficial for the reduction of the optical scattering loss [29]. The calculation of the cavity reflection spectrum is further carried out (Figure 4c), where multi‐mode emission is observed due to the relatively long cavity length of 1.08 µm. Considering that the spontaneous emission peak is located at over 285 nm (Figure 3b), a stimulated emission one at ∼285 nm can be expected.

Eventually, DUV VCSELs are optically pumped at room temperature by a 266‐nm laser with 5‐ns pulse duration and 20‐Hz repeat frequency (details in Figure S7). As the pump power increases, a stimulated emission peak with a linewidth of 0.11 nm is highlighted at 285.6 nm in Figure 4d, bringing about a *Q* factor of 2596. The dependence of the integrated intensity of the stimulated peak on the pump power density is further depicted (Figure 4e), where a clear kink (the threshold power density, P_th_) around 0.38 MW cm^−2^ is observed. The record low threshold in the DUV VCSELs is undoubtedly attributed to the precise control of the cavity length (state of the art of the threshold in optically pumped UV VCSELs in Figure S8) [8, 9, 21, 23, 40]; as a consequence, the resonance wavelength is perfectly matched with the gain peak (Figure S9). Besides, more dies across the 4‐in wafer are characterized to investigate the uniformity of lasing wavelength/cavity length, as shown in Figure 4f. Specifically, 4 random dies are picked up in each quadrant, and the lasing wavelength shifts in the range of 284.8–286.7 nm in the four quadrants (typical stimulated emission spectra in Figure S10), corresponding to a cavity length variation of 8.7 nm, ∼0.81% of the total length (Figure S11).

## 3 Conclusions

A DUV‐VCSEL strategy featuring the precise and uniform nanometer‐class control of the cavity length in the 4‐in wafer is demonstrated in the DUV framework based on GaN templates, which ensures the LLO removal of the sapphire substrates, and then provides space for the subsequent deposition of dielectric DBR. It is more significant that the strategy brings about a GaN/AlGaN sharp interface with an Al composition difference up to 80%, whereby self‐terminated etching with an ultrahigh selectivity of 100:1 is achieved for the exposed N‐polar GaN template relative to the Al_0.8_Ga_0.2_N pre‐crack layer after the sapphire removal. The cavity length, as a consequence, can be accurately determined by epitaxy instead of the fabrication process, which helps to minimize the detuning between the resonance wavelength and gain peak. Meanwhile, the etching brings about a smooth surface with the RMS roughness of 0.7 nm in an area of 3 × 3 µm^2^, efficiently reducing the optical scattering losses at the interface between the cavity and the top DBR. As such, a record low threshold of 0.38 MW cm^−2^ as well as a narrow linewidth of 0.11 nm is realized in the 285.6‐nm optically pumped DUV VCSELs with double dielectric DBRs. What's more, the lasing wavelength varies within 1.9 nm across the 4‐in wafer, corresponding to a cavity length variation of 8.7 nm, ∼0.81% of the total length of 1.08 µm. This work establishes a promising strategy for III‐nitride DUV VCSELs, which will greatly promote the development of this field and bring about devices featuring high performance and scalability.

## 4 Experimental Section

### 4.1 MOCVD Growth of the DUV VCSELs

The DUV‐VCSEL structures in this study were grown on the 4‐in double‐polished sapphire substrates by an Aixtron 1 × 4 in close‐coupled showerhead MOCVD system, and repeated by an AMEC Prismo HiT3 (4 × 4 in) MOCVD system. A 4‐µm‐thick GaN template was first grown by the two‐step method, followed by a 90‐nm‐thick Al_0.8_Ga_0.2_N pre‐crack layer and an 870‐nm‐thick Al_0.65_Ga_0.35_N healing layer grown at 1075°C and 1095°C, respectively. Then, 8‐pair 9‐nm/1.9‐nm Al_0.5_Ga_0.5_N/Al_0.37_Ga_0.63_N MQWs, as well as a 45‐nm‐thick Al_0.5_Ga_0.5_N capping layer, were grown in sequence.

### 4.2 Fabrication of the DUV VCSELs

A total of 8.5‐pair 35.5‐nm/40.8‐nm alternating HfO_2_/SiO_2_ layers were deposited on the as‐grown epitaxial surface as the bottom DBR. Then, the wafer was bonded to a Si submount, followed by the laser lift‐off process by employing a 248‐nm KrF excimer laser. After the removal of sapphire, the exposed N‐polar GaN surface was cleaned in the HCl solution for 1 min to remove the Ga metal from GaN decomposition. The residual GaN layer was then etched down to the Al_0.8_Ga_0.2_N pre‐crack layer by ICP. Eventually, 7.5‐pair HfO_2_/SiO_2_ was deposited on the N‐polar surface as the top DBR. The SEM image of the DUV VCSELs is shown in Figure S12.

### 4.3 Characterization

TEM (Thermo Scientific Themis Z STEM operated at 200 kV) was employed to reveal the control of cavity length in this study, where the TEM specimen was prepared by focused ion beam (FIB, Thermo Scientific Helios G4 HX Dual Beam). The surface morphology of wafers was evaluated by AFM (Bruker Dimension Icon) and SEM (FEI Nova NanoSEM 430). Reflection and transmission spectra were recorded by Agilent Cary 7000 UV−vis−NIR spectrophotometry. Temperature‐dependent PL was characterized by a lab‐made system at Peking University, where a 213‐nm laser (Xiton Photonics Impress 213) was employed as the excitation source. Temperature‐dependent measurements were performed by employing a closed‐cycle helium cryostat (JANIS SVT‐400) attached to the temperature controller (Scientific Instruments 9700). Time‐resolved as well as polarization‐dependent PL was characterized by a lab‐made system at Peking University, where a 266‐nm laser (Coherent Chameleon Ultra II) was employed as the excitation source, and a streak camera (Hamamatsu C10910‐03) and a spectrometer (Horiba iHR550) were used for time‐resolved and polarization‐dependent detection, respectively. The optical pump of the DUV VCSELs was performed by a lab‐made system at Xiamen University, and a 266‐nm laser with 5‐ns pulse duration and 20‐Hz repeat frequency was employed as the pump source.

## Author Contributions

C.J. and J.M.W. conceived the experiments. C.J. and J.M.W. grew the samples. C.J., C.Z.J., J.Z., and E.Z. performed relevant measurements. F.X., L.Z., X.Y., N.T., X.W., W.G., and B.S. gave support in the measurements and analyses. C.J., J.M.W., and J.L. performed device fabrication under X.K., Z.Q., and J.J.W. supervision. J.M.W. wrote the manuscript with the assistance of F.X., W.G., and B.S. All authors discussed the results and commented on the manuscript.

## Conflicts of Interest

The authors declare no conflict of interest.

## Supporting information




**Supporting File**: advs73432‐sup‐0001‐SuppMat.docx

## Data Availability

The data that support the findings of this study are available from the corresponding author upon reasonable request.
