# Supplementary material for: Wafer Scale III‐Nitride Deep‐Ultraviolet Vertical‐Cavity Surface‐Emitting Lasers Featuring Nanometer‐Class Control of Cavity Length
Source: Adv Sci (Weinh). 2025 Dec 21;13(13):e20405. doi: 10.1002/advs.202520405 (PMC12955920; doi:10.1002/advs.202520405)
Supplement: Supplementary file 1 — Supporting File: advs73432‐sup‐0001‐SuppMat.docx [file ADVS-13-e20405-s001.docx]

Supporting Information

Wafer Scale III-Nitride Deep-Ultraviolet Vertical-Cavity Surface-Emitting Lasers Featuring Nanometer-Class Control of Cavity Length

Chen Ji, Jiaming Wang^*^, Fujun Xu^*^, Lisheng Zhang, Jing Lang, Ziyao Zhang, Fuyun Tan, Chengzhi Ji, Junchuan Zhang, Erfei Zhang, Xiangning Kang, Zhixin Qin, Guangxu Ju, Jiejun Wu, Xuelin Yang, Ning Tang, Xinqiang Wang, Weikun Ge, and Bo Shen^*^

**Table of contents**

**Figure S1.** Transmission spectra of the top and bottom DBRs.

**Figure S2.** Variation of the Al composition at the interface between the GaN template and Al_0.8_Ga_0.2_N pre-crack layer.

**Figure S3.** The origin of small protrusions after the self-terminated etching.

**Figure S4.** Bowing of the as-grown and processed wafers.

**Figure S5.** Raman spectra of E_2_ (high) phonon mode of the GaN templates in the as-grown and processed wafers.

**Figure S6.** Cross-sectional SEM image of the DUV VCSELs.

**Figure S7.** Estimation of pump power density in consideration of the transmission through the top DBR at 266 nm.

**Figure S8.** State of the art of the threshold power density in optically pumped UV VCSELs with the wavelength shorter than 400 nm.

**Figure S9.** The wavelength difference between the stimulated and spontaneous peaks.

**Figure S10.** Typical stimulated emission spectra in the four quadrants of the 4-in wafer.

**Figure S11.** Calculated cavity reflection spectra for the lasing wavelength of 284.8 and 286.7 nm, respectively.

**Figure S12.** Plan-view SEM image of the DUV-VCSEL array.


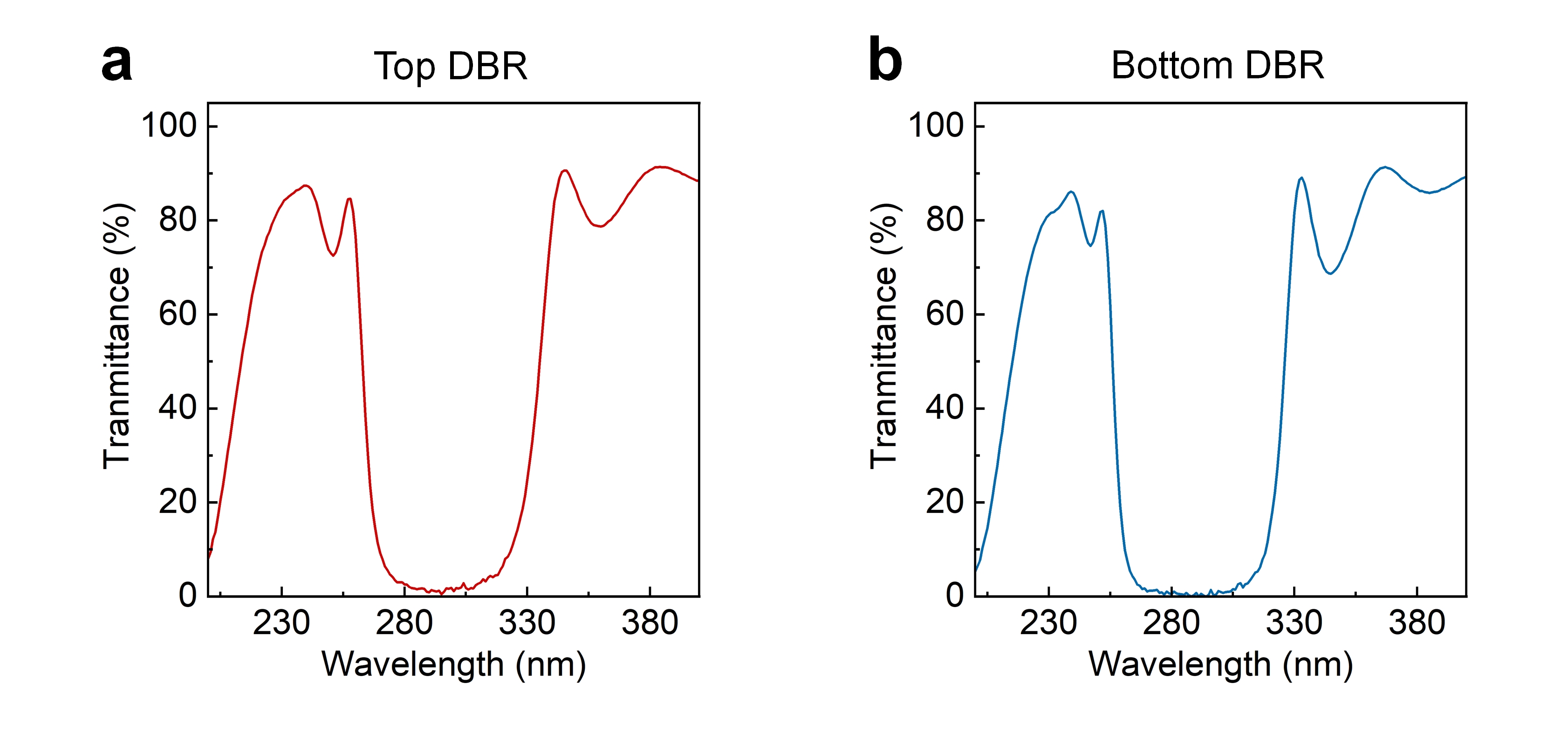


**Figure S1.** Transmission spectra of the top and bottom DBRs. a) The top DBR. b) The bottom DBR.

The transmission spectra of the top and bottom DBRs at normal incidence are depicted in Figure S1a and S1b, respectively, where the stopband widths reach ~70 nm for both spectra. The transmissivity is 1.55% and 0.36% at 285 nm in the top and bottom DBRs, respectively. The slight inconsistency between the transmissivity and corresponding reflectivity (in Figure 1e and 1f) is attributed to the absorption inside the DBRs.


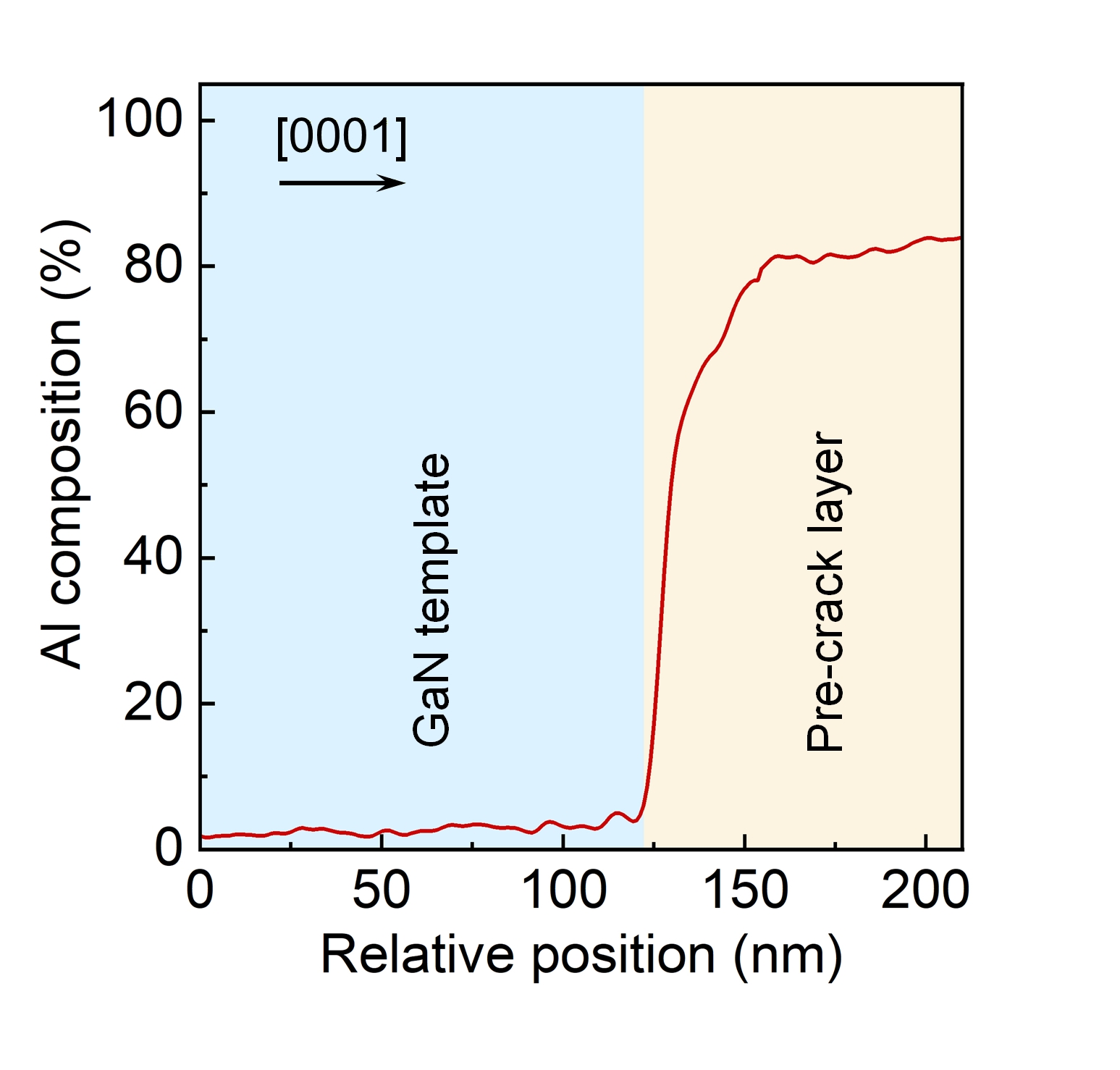


**Figure S2.** Variation of the Al composition (EDS) at the interface between the GaN template and Al_0.8_Ga_0.2_N pre-crack layer.


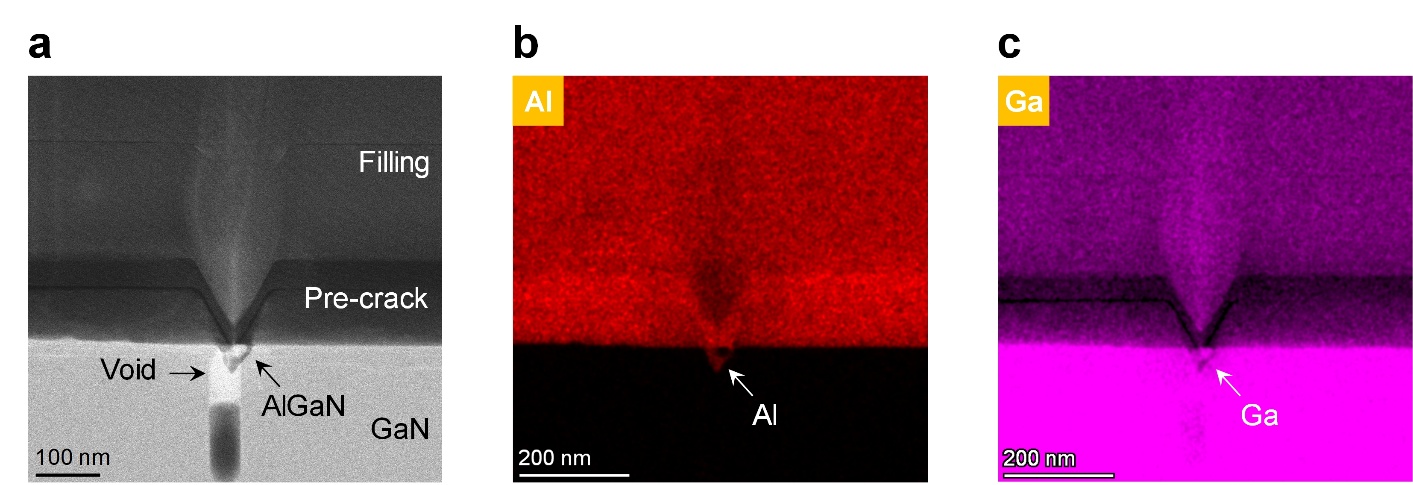


**Figure S3.** The origin of small protrusions after the self-terminated etching. a) Cross-sectional HAADF-STEM image of the filled-up pre-crack. b,c) Distribution of Al and Ga atoms around the pre-crack in Panel a. Reproduced with permission.^[1]^ Copyright 2024, Springer Nature.

Details about the healing of pre-cracks in the DUV framework based on GaN templates were described in our previous research.^[1]^ It has been found that the tensile strain-induced cracks run through the Al_0.8_Ga_0.2_N pre-crack layer, and even propagate to the GaN template, resulting in the voids as show in Figure S3a. In the subsequent growth of the healing layer, the pre-cracks are filled, while the voids in GaN are reserved. It should be noted that the void in Panel a is partially filled, in particular around the interface between the pre-crack layer and GaN template, since there are evident Al and Ga signals inside the void according to the EDS mapping measurements in Figure S3b and S3c, respectively. That explains the origin of the small protrusions after the self-terminated etching in Figure 2f.


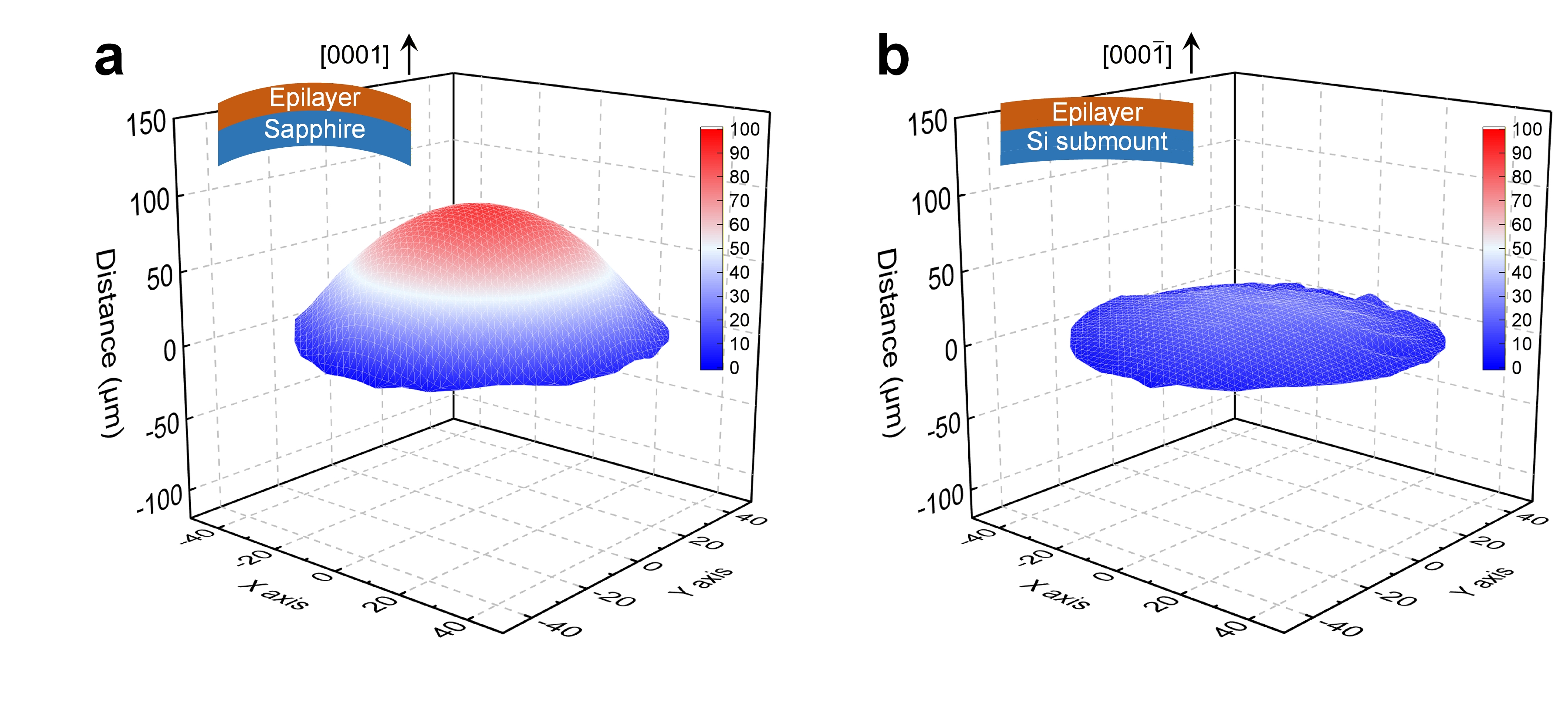


**Figure S4.** Bowing of the as-grown and processed wafers. a) The as-grown wafer. b) The processed wafer after bonding and LLO.

According to the Stoney’s equation, the residual stress in the epilayer is proportional to the curvature.^[2]^ Consequently, the bow values of the 4-in as-grown and processed (bonding and LLO) wafers are measured (EtaMax Plato) to reveal the variation of the residual stress owing to the processes of bonding and LLO, as shown in Figure S4a and S4b, respectively. Convex bending can be observed in both wafers, where the bow values are 90 and 25 μm in the as-grown and processed wafer, respectively. In other words, the residual stress is released in the processes of bonding and LLO.


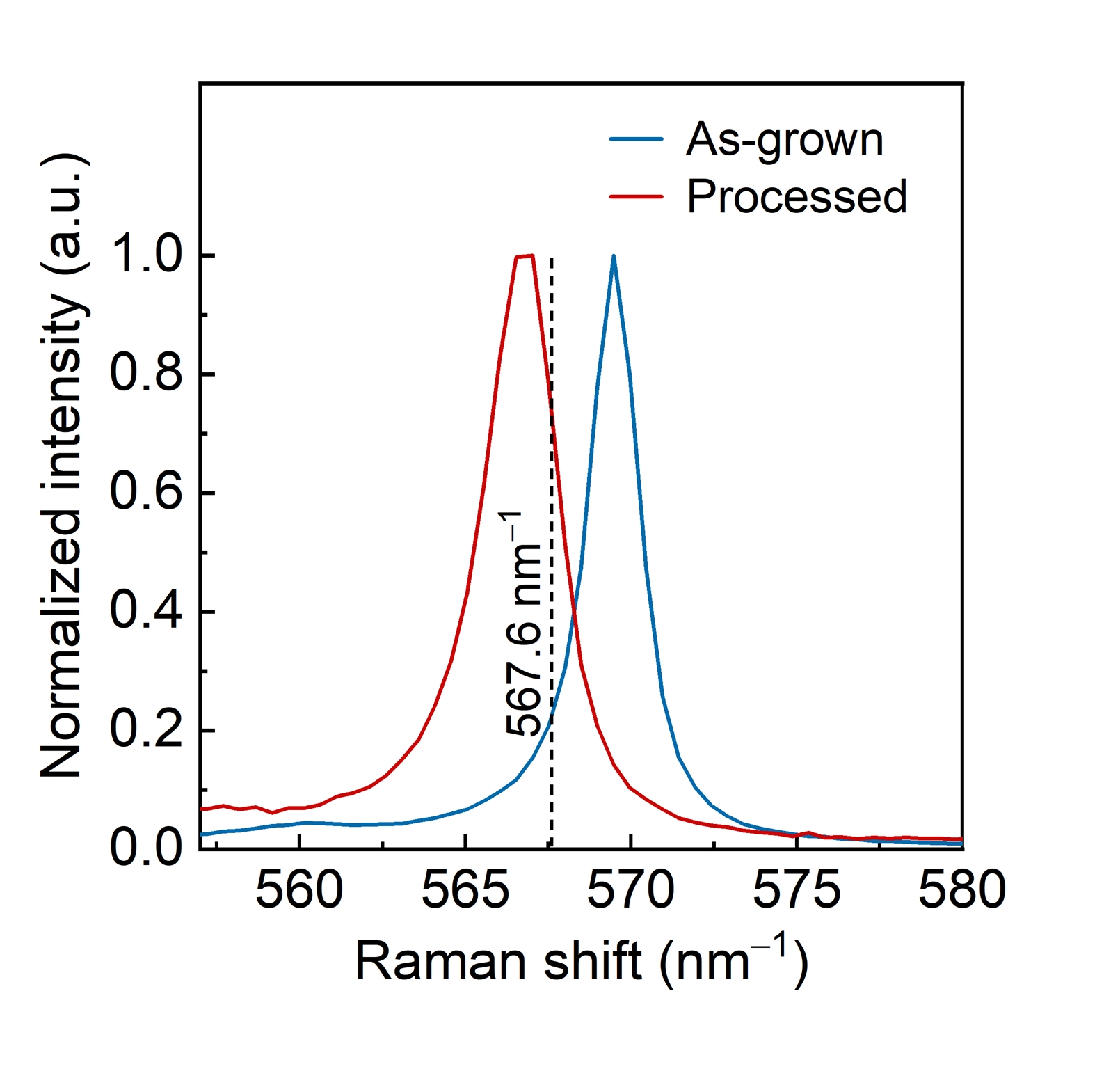


**Figure S5.** Raman spectra of E_2_ (high) phonon mode of the GaN templates in the as-grown and processed wafers.

The stress in GaN templates is modulated after the processes of bonding and LLO, according to the Raman shift in Figure S5. A black dash line is plotted at 567.6 cm^−1^, corresponding to the theoretical position of E_2_ (high) phonon mode in freestanding GaN.^[3]^ It is found that the E_2_ (high) peak in the as-grown wafer locates at 569.5 cm^−1^, indicating a compressive stress in the GaN template, consistent with the convex shape in Figure S4a. While the stress is released in the processed wafer, where a tensile stress in the GaN template is even demonstrated.


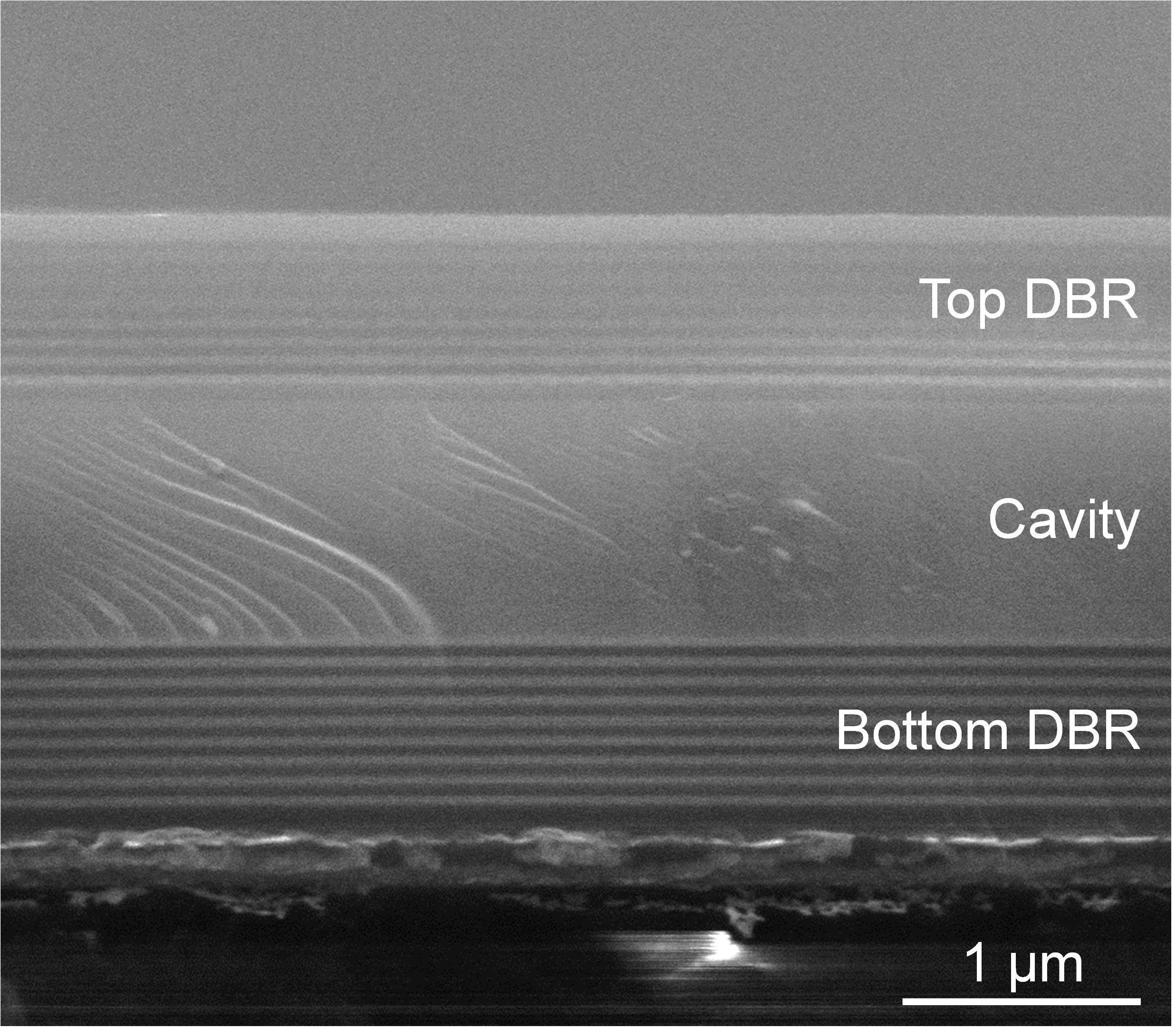


**Figure S6.** Cross-sectional SEM image of DUV VCSELs.


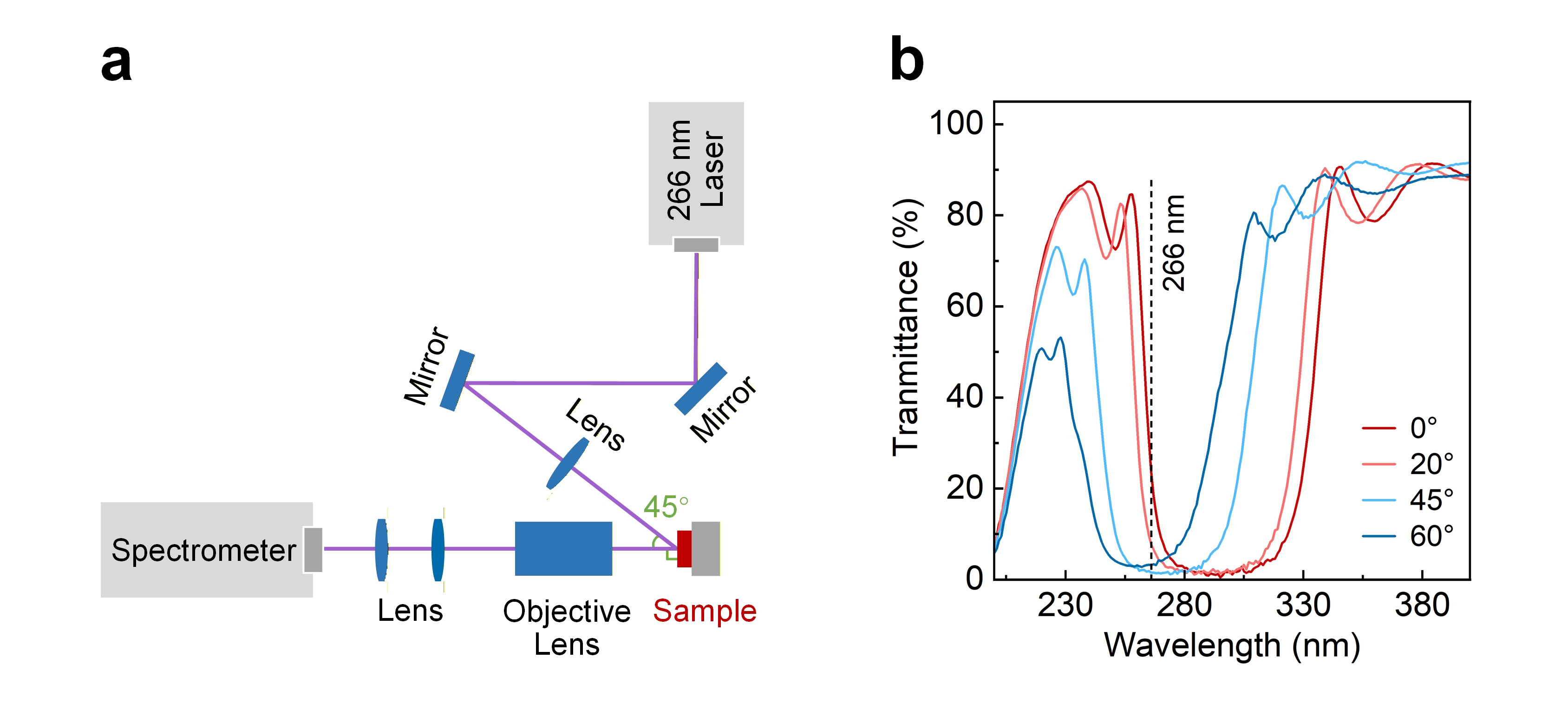


**Figure S7.** Estimation of pump power density in consideration of the transmission through the top DBR at 266 nm. a) Schematic illustration of the optical configuration for the optically pumping measurements. b) Transmission spectra of the top DBR measured at different incidence angles.

The 266-nm pump laser in this study is inevitably reflected and absorbed by the top DBR. Therefore, the effective pump power density shown in Figure 4d and 4e is calculated by considering the transmission of the top DBR at 266 nm. Figure S7a shows the schematic illustration of the optical configuration for pumping, where the laser is incident obliquely with an angle of 45°. The transmission spectra of the top DBR are then measured at different incidence angles (Figure S7b), and evident spectral shift with respect to the angle can be observed. Finally, the transmittance for the 266-nm laser is determined to be 1.56% with an incidence angle of 45°. The pump power density in the main manuscript is calculated by the laser power density in front of the top DBR multiplying this transmittance of 1.56%.


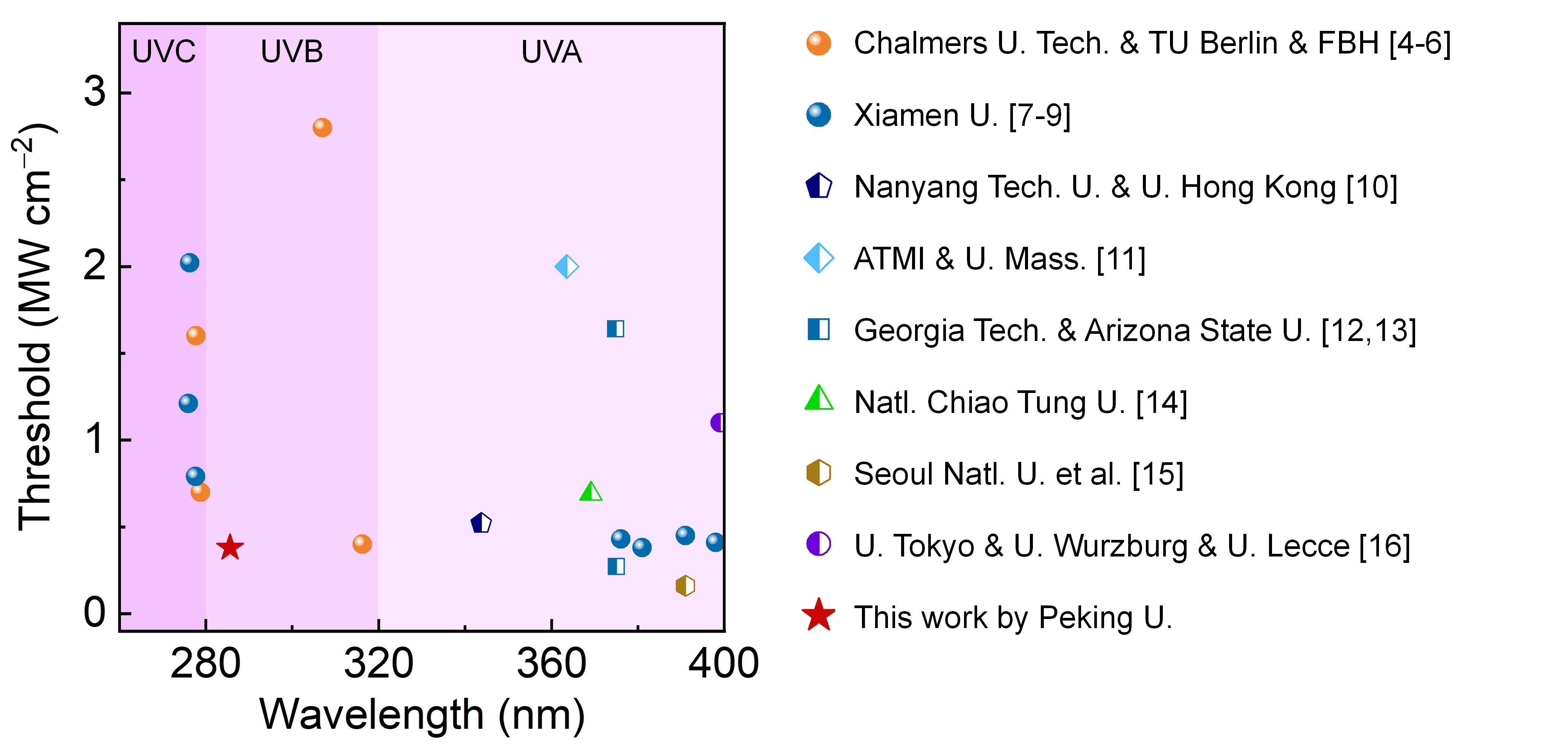


**Figure S8.** State of the art of the threshold power density in optically pumped UV VCSELs with the wavelength shorter than 400 nm.


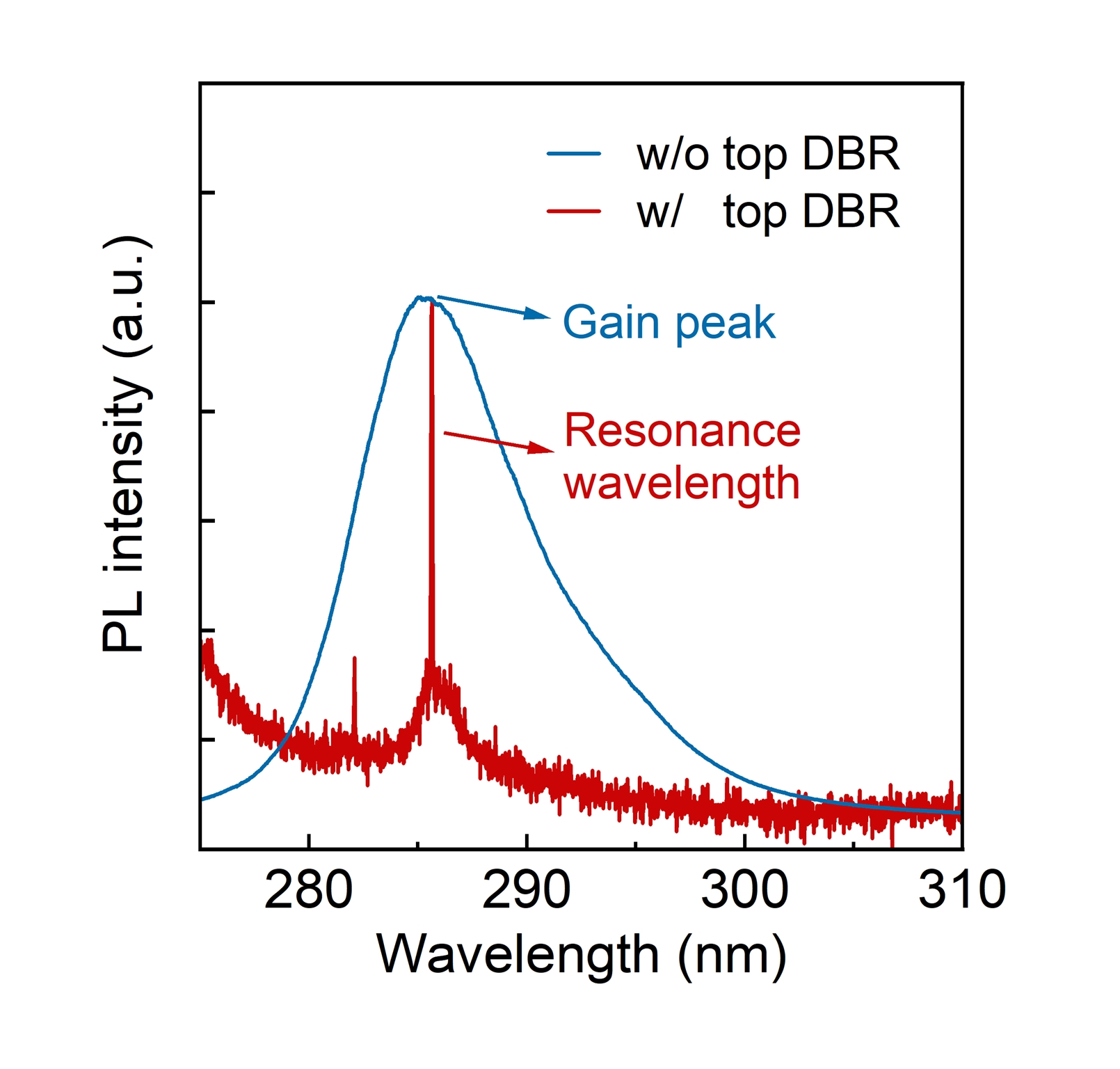


**Figure S9.** The detuning between the resonance wavelength and gain peak.

In order to study the detuning between the resonance wavelength and gain peak, the room-temperature PL spectra of the processed MQWs is performed at as the threshold pump power density shown in Figure S9, before the deposition of the top DBR. It is found than the gain peak is perfectly matched with resonance wavelength (285.6 nm), leading to a low threshold.


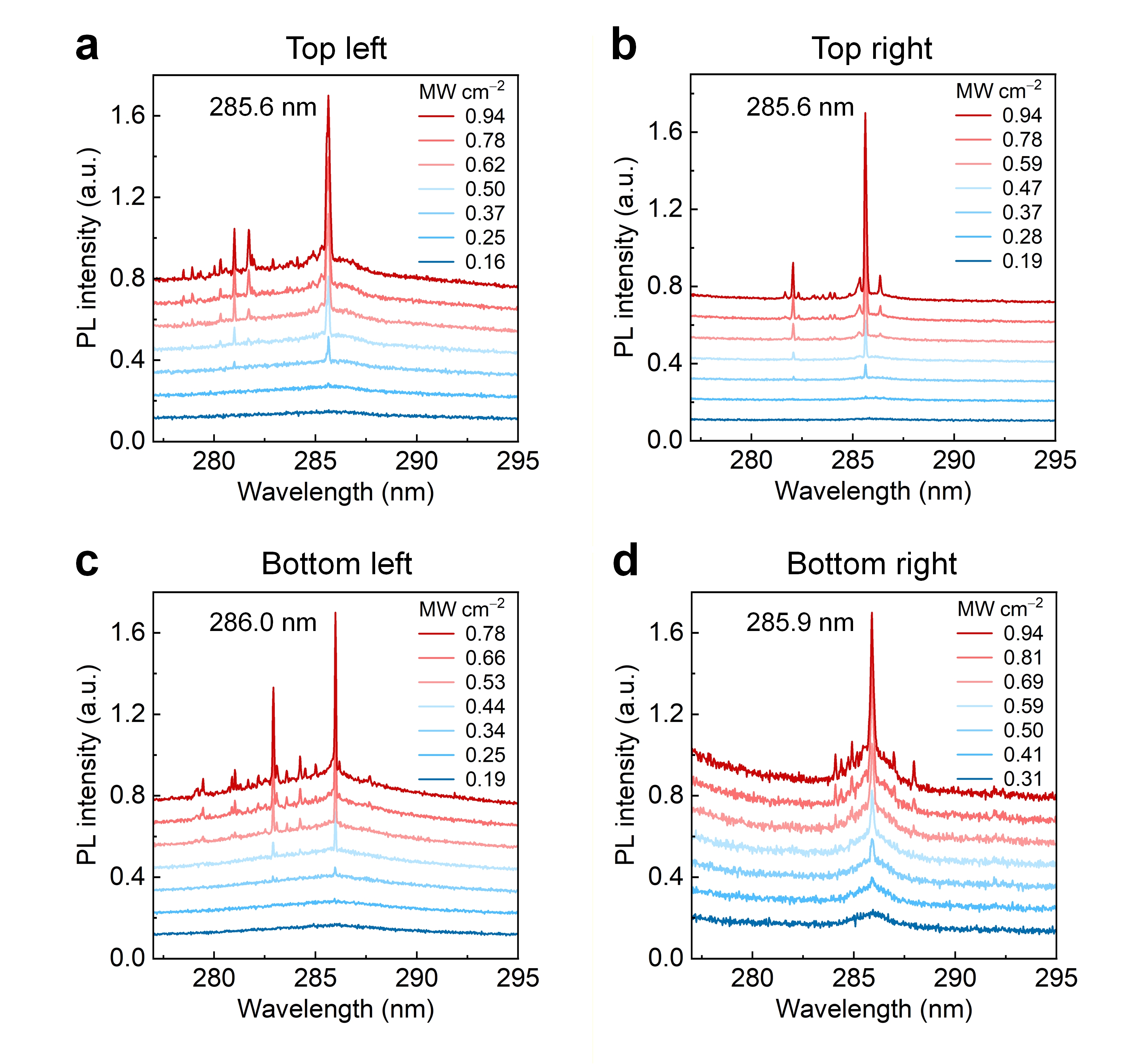


**Figure S10.** Typical stimulated emission spectra in the four quadrants of the 4-in wafer. a) Top left. b) Top right. c) Bottom left. d) Bottom right.


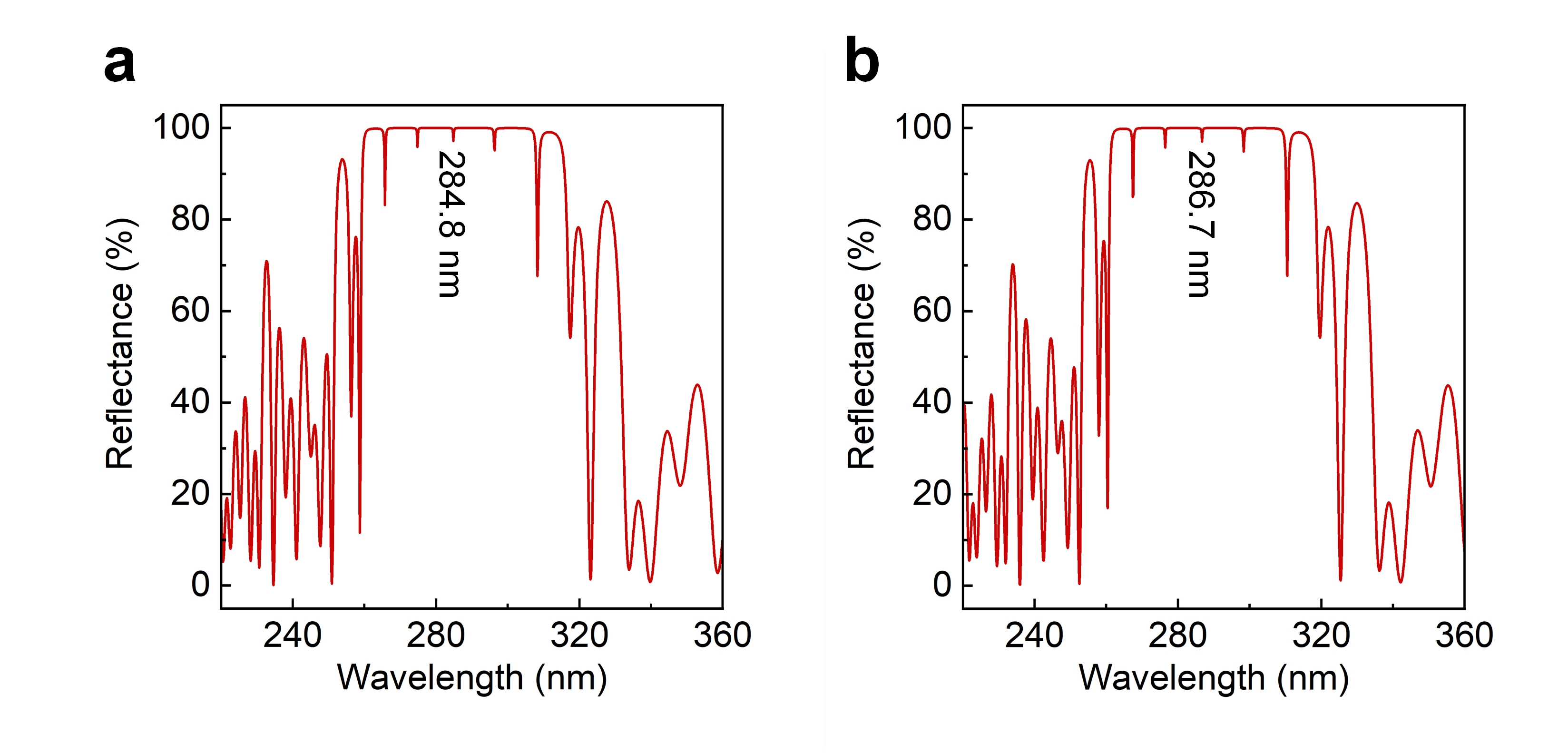


**Figure S11.** Calculated cavity reflection spectra for different lasing wavelengths. a) 284.8 nm. b) 286.7 nm.

It is convinced that the etching depth difference of the Al_0.8_Ga_0.2_N pre-crack layer across the 4-in wafer should be primarily responsible for the uniformity of the lasing wavelength. Herein the cavity reflection spectra are calculated by only varying the Al_0.8_Ga_0.2_N thickness. It is found that the lasing wavelength of 284.8 and 286.7 nm corresponds to the Al_0.8_Ga_0.2_N thickness of 78.5 and 87.2 nm, respectively. In other words, there is a cavity length variation of 8.7 nm, ~0.81% of the total length.


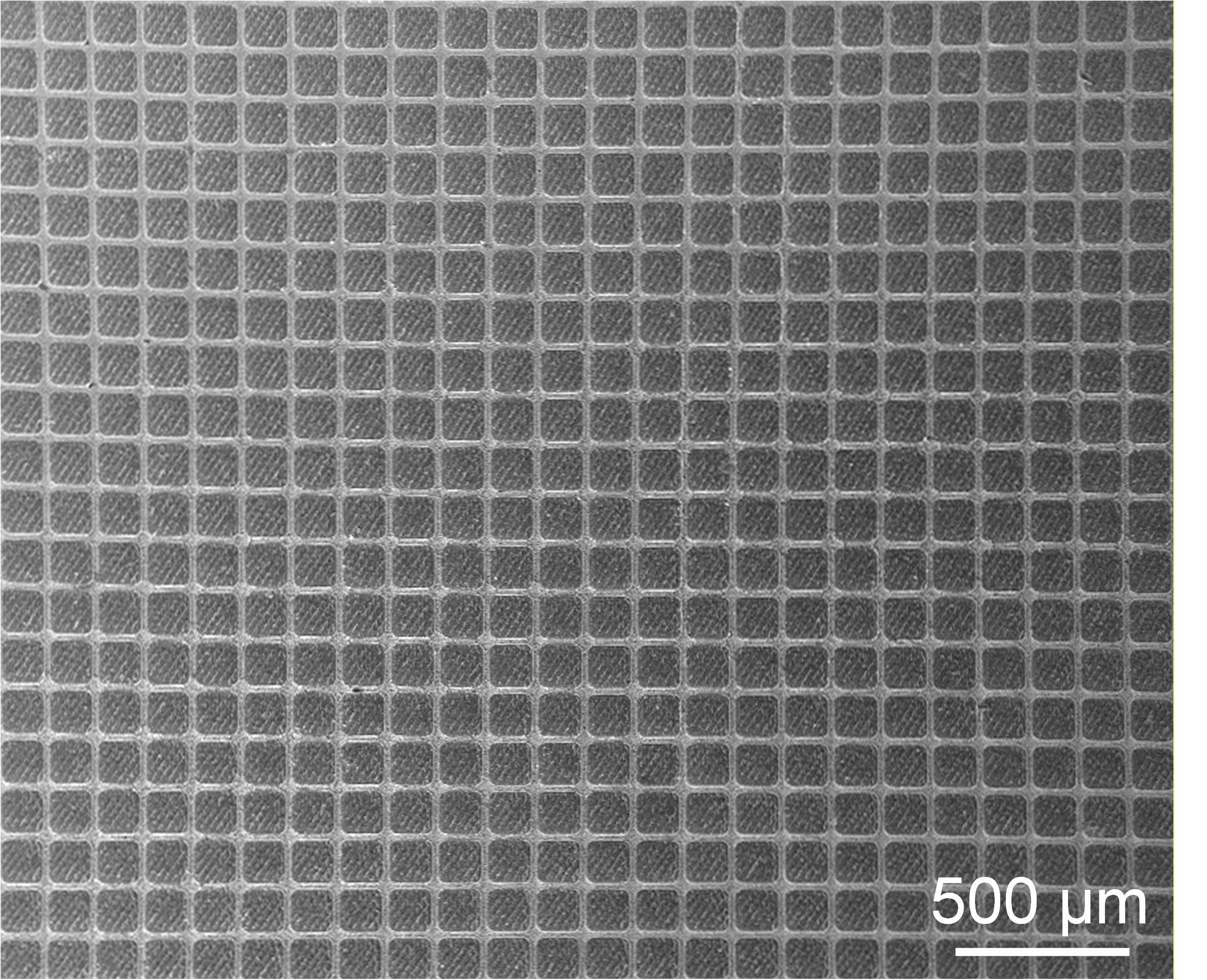


**Figure S12.** Plan-view SEM image of the DUV-VCSEL array.

Figure S12 presents the plan-view SEM image of the DUV-VCSEL array, where the size of the devices is designed as 130×130 μm^2^, with an interval of 12 μm.

**References**

1. J. Wang, C. Ji, J. Lang, F. Xu, L. Zhang, X. Kang, Z. Qin, X. Yang, N. Tang, X. Wang, W. Ge, and B. Shen, Wafer-scale vertical injection III-nitride deep-ultraviolet light emitters. *Nature Communications* **2024**, 15, 9398.
2. T.-L. Chou, S.-Y. Yang, and K.-N. Chiang, Overview and applicability of residual stress estimation of film–substrate structure. *Thin Solid Films* **2011**, 519, 7883.
3. V. Y. Davydov, Y. E. Kitaev, I. N. Goncharuk, A. N. Smirnov, J. Graul, O. Semchinova, D. Uffmann, M. B. Smirnov, A. P. Mirgorodsky, and R. A. Evarestov, Phonon dispersion and Raman scattering in hexagonal GaN and AlN. *Physical Review B* **1998**, 58, 12899.
4. E. Torres, J. Ciers, N. Rebelo, F. Hjort, M. A. Bergmann, S. Graupeter, J. Enslin, G. Cardinalli, T. Wernicke, M. Kneissl, and Å. Haglund, Ultraviolet-C vertical-cavity surface-emitting lasers with precise cavity length control. *Laser & Photonics Reviews* **2025**, 19, 2402203.
5. F. Hjort, J. Enslin, M. Cobet, M. A. Bergmann, J. Gustavsson, T. Kolbe, A. Knauer, F. Nippert, I. Häusler, M. R. Wagner, T. Wernicke, M. Kneissl, and Å. Haglund, A 310 nm optically pumped AlGaN vertical-cavity surface-emitting laser. *ACS Photonics* **2021**, 8 135.
6. G. Cardinali, F. Hjort, N. Prokop, J. Enslin, M. Cobet, M. A. Bergmann, J. Gustavsson, J. Ciers, I. Häusler, T. Kolbe, T. Wernicke, Å. Haglund, and M. Kneissl, Low-threshold AlGaN-based UVB VCSELs enabled by post-growth cavity detuning. *Applied Physics Letters* **2022**, 121, 103501.
7. Z. Zheng, Y. Mei, H. Long, J. Hoo, S. Guo, Q. Li, L. Ying, Z. Zheng, and B. Zhang, AlGaN-based deep ultraviolet vertical-cavity surface-emitting laser. *IEEE Electron Device Letters* **2021**, 42 375.
8. Z. Zheng, Y. Wang, J. Hoo, S. Guo, Y. Mei, H. Long, L. Ying, Z. Zheng, and B. Zhang, High-quality AlGaN epitaxial structures and realization of UVC vertical-cavity surface-emitting lasers. *Science China Materials* **2023**, 66, 1978.
9. Y. Mei, T.-R. Yang, W. Ou, Z.-M. Zheng, H. Long, L.-Y. Ying, and B.-P. Zhang, Low-threshold wavelength-tunable ultraviolet vertical-cavity surface-emitting lasers from 376 to 409 nm. *Fundamental Research* **2021**, 1, 684.
10. R. Chen, H. D. Sun, T. Wang, K. N. Hui, and H. W. Choi, Optically pumped ultraviolet lasing from nitride nanopillars at room temperature. *Applied Physics Letters* **2010**, 96, 241101.
11. J. M. Redwing, D. A. S. Loeber, N. G. Anderson, M. A. Tischler, and J. S. Flynn, An optically pumped GaN–AlGaN vertical cavity surface emitting laser. *Applied Physics Letters* **1996**, 69, 1.
12. Y.-S. Liu, A. F. M. Saniul Haq, K. Mehta, T.-T. Kao, S. Wang, H. Xie, S.-C. Shen, P. D. Yoder, F. A. Ponce, T. Detchprohm, and R. D. Dupuis, Optically pumped vertical-cavity surface-emitting laser at 374.9 nm with an electrically conducting n-type distributed Bragg reflector. *Applied Physics Express* **2016**, 9, 111002.
13. Y. J. Park, T. Detchprohm, K. Mehta, J. Wang, H. Jeong, Y.-S. Liu, P. Chen, S. Wang, S.-C. Shen, P. D. Yoder, F. Ponce, and R. D. Dupuis, Optically pumped vertical-cavity surface-emitting lasers at 375 nm with air-gap/Al_0.05_Ga_0.95_N distributed Bragg reflectors. *Proceedings of SPIE* **2019**, 10938, 109380A.
14. T.-C. Chang, S.-Y. Kuo, E. Hashemi, Å. Haglund, and T.-C. Lu, GaN vertical-cavity surface-emitting laser with a high-contrast grating reflector. *Proceedings of SPIE* **2018**, 10542, 105420T.
15. S.-H. Park, J. Kim, H. Jeon, T. Sakong, S.-N. Lee, S. Chae, Y. Park, C.-H. Jeong, G.-Y. Yeom, and Y.-H. Cho, Room-temperature GaN vertical-cavity surface-emitting laser operation in an extended cavity scheme. *Applied Physics Letters* **2003**, 83, 2121.
16. T. Someya, R. Werner, A. Forchel, M. Catalano, R. Cingolani, and Y. Arakawa, Room temperature lasing at blue wavelengths in gallium nitride microcavities. *Science* **1999**, 285, 1905.
